# Quercetin- and Rutin-Containing Electrospun Cellulose Acetate and Polyethylene Glycol Fibers with Antioxidant and Anticancer Properties

**DOI:** 10.3390/polym14245380

**Published:** 2022-12-08

**Authors:** Nikoleta Stoyanova, Mariya Spasova, Nevena Manolova, Iliya Rashkov, Ani Georgieva, Reneta Toshkova

**Affiliations:** 1 Laboratory of Bioactive Polymers, Institute of Polymers, Bulgarian Academy of Sciences, Acad. G. Bonchev St, bl. 103A, BG-1113 Sofia, Bulgaria; 2 Institute of Experimental Morphology, Pathology and Anthropology with Museum, Bulgarian Academy of Sciences, Acad. G. Bonchev St, bl. 25, BG-1113 Sofia, Bulgaria

**Keywords:** flavonoids, electrospinning, cellulose derivative, water-soluble polyether, antioxidant activity, HeLa, mouse BALB/c 3T3 fibroblasts

## Abstract

Innovative fibrous materials from cellulose derivative, cellulose acetate (CA) and water-soluble polyether, polyethylene glycol (PEG) loaded with natural biologically active compounds (BAC), quercetin (QUE) and rutin (RUT), have been successfully fabricated by blend electrospinning and dual electrospinning. Scanning electron microscopy revealed that the mean fiber diameters of all the obtained fibers were in the nanometer range. QUE and RUT incorporated in the fibrous mats were in the amorphous state, as evidenced by the performed differential scanning calorimetry (DSC) and X-ray diffraction (XRD) analysis. The presence of the polyether in the developed fibrous material assisted the in vitro release of the biologically active compounds by improving the hydrophilicity and wettability of the mats. Rutin-containing fibrous materials manifest the highest antioxidative activity, as determined by the 2,2-diphenyl-1-picryl-hydrazyl-hydrate free radical method. The cytotoxicity of the fabricated novel materials was evaluated using a tumor cell line and normal mouse fibroblast cells. The mats containing QUE and QUE/RUT independent of the applied spinning method show a higher cytotoxic effect against cancer cells and 3 to 4.5 times lower cytotoxicity to a noncancer cell line. These features make the quercetin- and rutin-containing fibrous materials promising candidates for pharmaceutical, cosmetic, and biomedical use.

## 1. Introduction

The naturally occurring polyphenolic compounds known as flavonoids may be found in a variety of foods, including red wine, chocolate, green tea, olive oil, bee propolis, fruits, vegetables, grains, bark, roots, stems, and flowers. The positive impacts of flavonoids on health are ascribed to their antioxidative, anti-inflammatory, antimutagenic, and anticarcinogenic characteristics [1]. Studies reveal that flavonoids have a protective effect against UV radiation [2], cardiovascular diseases [3], cancer [4,5,6], and other chronic diseases.

Rutin is a kind of flavonoid, also known as vitamin P. It was discovered in more Answer:than 70 plant species and food of plant origin, including buckwheat [7], apricots, cherries, grapes, grapefruit, onion, plums, and oranges [8,9]. RUT is a glycoside that chemically combines the flavonol quercetin with the disaccharide rutinose (rhamnose and glucose) [10]. This flavonoid exhibits a number of pharmacological properties [11,12,13,14,15], which have been related to its capacity as free radical scavengers [16,17].

The most prevalent flavonoid, quercetin, is very desirable for human nutrition and functional food. This naturally occurring compound belongs to the class called flavonols that cannot be produced in the human body. Quercetin exhibits antioxidant activity in addition to antiviral, anti-inflammatory, antibacterial, anticancer, and muscle-relaxing effects [18,19,20,21].

However, the main drawback of rutin and quercetin is their low bioavailability, which is mostly caused by their poor water solubility: 0.13 mg/mL for rutin and 0.01 mg/mL for quercetin [22,23].

The creation of appropriate polymer-based carriers able to increase the solubility of these flavonoids in aqueous environments is an effective method of improving the bioavailability of both compounds. Loading of biologically active compounds in an amorphous polymer matrix is a method to overcome the limited water solubility. Nowadays, electrospinning has emerged as a novel method for creating polymer drug delivery carriers [24]. This is because of the fact that when the diameters of the polymer fibers decrease to micrometers or nanometers interesting properties of the materials such as increase in specific surface area, potential for surface modification and modulation of the release profile of the incorporated biologically active compound occur which improves the therapeutic effect and mechanical properties, and decreases side effects. It has been demonstrated that bioactive molecules of both natural [25] and synthetic origin may be loaded effectively in electrospun materials [26].

Electrospinning is applied for the fabrication of rutin-loaded fibrous membranes based on cellulose acetate and polyethylene oxide. The surface structure, encapsulation effectiveness, antioxidant activity, antibacterial activity, and drug release of the rutin-loaded electrospun membranes were investigated. The study reveals that rutin-loaded fibrous membranes had 98.3% antioxidant activity and 93.5% and 95.0% antibacterial activity against *E. coli* and *S. aureus*, respectively [27]. By electrospinning, rutin-Pluronic solid dispersions were incorporated into nanofibers based on pullulan. According to XRD and DSC studies, loaded rutin is in an amorphous form and exhibits a burst release and similar antioxidant efficacy to crude rutin [28]. Electrospun polymer fibers composed of polycaprolactone, polylactic acid, polylactic-*co*-glycolic acid, poly (vinyl pyrrolidone), ethyl cellulose, zein, and cellulose acetate (CA)/PEG have been embedded with quercetin [29,30,31,32,33].

Recently, electrospun chitosan oligosaccharide/polycaprolactone nanofibers loaded with rutin and quercetin were obtained for antibacterial applications [34]. However, their anticancer properties have not been studied.

Therefore, the aim and the novelty of the present study consist in finding suitable conditions for incorporating the combinations of QUE and RUT into micro- and nanofibrous materials by electrospinning and analyzing their physicochemical and biological characteristics. In order to enhance the release of both active compounds from fibers, the polymer matrix’s composition was carefully selected. Moreover, the effect of the substituent in the structure of the biologically active compound on their antioxidant and anticancer activities is studied.

## 2. Materials and Methods

### 2.1. Used Materials

In the study, quercetin (QUE, 95%; Sigma-Aldrich, St. Louis, MO, USA), rutin (RUT, 97+%; Acros Organics, Geel, Belgium), polyethylene glycol (PEG; Fluka, Buchs, Switzerland), cellulose acetate (CA; Aldrich, St. Louis, MO, USA) with = 30,000 g/mol, and Tween 80 (Acros Organics, Geel, Belgium) were used. Both acetone and ethanol were of analytical grade (Sigma-Aldrich, Darmstadt, Germany) and were used as supplied. 2,2-Diphenyl-1-picrylhydrazyl (DPPH) from Sigma-Aldrich (Darmstadt, Germany), 3-(4.5-dimethylthiazol-2-yl)-2.5-diphenyltetrazolium bromide (MTT; Sigma-Aldrich, Darmstadt, Germany), ethidium bromide (EtBr; Sigma Chemical, Balcatta, WA, Australia), acridine orange (AO; Sigma Chemical, Balcatta, WA, Australia), and 4’,6-diamidino-2-phenylindole dihydrochloride (DAPI; Sigma-Aldrich Darmstadt, Germany) were utilized without additional purification as they were of analytical grade of purity. The antibiotics penicillin and streptomycin (Lonza, Cologne, Germany) and fetal calf serum (FCS; Gibco, Wien, Austria) were added to Dulbecco’s Modified Eagle’s Medium (DMEM; Sigma-Aldrich, Darmstadt, Germany).

### 2.2. Electrospinning Technique for Fabrication of Fibrous Mats

Five various types of fibrous materials, including CA/PEG, CA/PEG/QUE, CA/PEG/RUT, and CA/PEG/QUE/RUT, were made by electrospinning. One complex fibrous material was prepared by employing dual electrospinning of CA/PEG/QUE solution and CA/PEG/RUT solution to obtain the effect of varied architecture. For performing electrospinning, the following spinning solutions were obtained in a mixture of acetone/water 80/20 *v/v*: (1) CA/PEG (80/20 *w*/*w*), (2) CA/PEG (80/20 *w*/*w*) with QUE (10 wt% to the polymer weight), (3) CA/PEG (80/20 *w*/*w*) with RUT (10 wt% to the polymer weight), (4) CA/PEG (80/20 *w*/*w*) with QUE (5 wt% to the polymer weight), and RUT (5 wt% to the polymer weight) for a blend solution as well as (5) CA/PEG (80/20 *w*/*w*) with RUT (5 wt% to the polymer weight) and CA/PEG (80/20 *w*/*w*) with QUE (5 wt% to the polymer weight) for a dual electrospinning experiment and (6) CA/PEG (80/20 *w*/*w*) with QUE (5 wt% to the polymer weight) for a dual electrospinning experiment. The total polymer concentration was 10 wt%. The as-prepared solutions were then transferred into a 5 mL syringe equipped with a metal needle (size: 20GX1½″) whose tip was attached to the positively charged electrode. The electrode was connected to a specially constructed high-voltage power source that could generate positive DC voltages between 10 and 30 kV. The grounded rotating drum, which had a diameter of 45 mm, was positioned 15 cm away from the needle’s tip. The collector rotation speed was 1000 rpm. An infusion pump (NE-300 Just Infusion^TM^ Syringe Pump, New Era Pump Systems Inc., Farmingdale, NY, USA) was used to deliver the spinning solution at a feed rate of 3 mL/h, a constant applied voltage of 25 kV, a room temperature of 21 °C, and a relative humidity of 52%.

For the dual electrospinning, two infusion pumps (NE-300 Just Infusion^TM^ Syringe Pump, New Era Pump Systems Inc., Farmingdale, NY, USA), positioned on either side of the collector at an angle of 180°, were supplied with syringes containing the CA/PEG/RUT and CA/PEG/QUE solutions. An amount of 3.0 mL/h was the feed rate used to deliver both solutions. The applied voltage was 25 kV, and the tip-to-collector distance was 15 cm. To eliminate any remaining solvent, all the prepared fibrous mats were placed under reduced pressure at 25 °C.

### 2.3. Complex Characterization of the Fibrous Materials

Using scanning electron microscopy, the produced fibrous materials underwent a detailed morphological investigation (SEM). All the mats were vacuum-coated with gold for 60 s prior to observation in a Jeol JFC-1200 fine coater, and were then studied using a Jeol JSM-5510 (Jeol Co., Ltd., Tokyo, Japan). At least 30 fibers from SEM images were measured using the ImageJ software to determine the mean fiber diameter and the standard deviation [35].

An IRAffinity-1 spectrophotometer (Shimadzu, Kyoto, Japan) equipped with a MIRacle ATR accessory (diamond crystal; the depth of penetration of the IR beam into the material is 2 μm) was used to perform attenuated total reflection Fourier-transform infrared (ATR-FTIR) spectroscopic investigations. A DLATGS detector connected with a temperature controller was used to record the spectra, which ranged from 4000 to 500 cm^−1^ with a spectral resolution of 4 cm^−1^. Using IRsolution’s software, the H_2_O and CO_2_ content of all spectra was adjusted. Prior to analysis, all samples were dried under reduced pressure.

For analyzing the crystalline structure of the fabricated fibrous materials, X-ray diffraction analysis (XRD) was carried out. The XRD patterns were recorded using a D8 Bruker Advance powder diffractometer (Bruker, Billerica, MA, USA) equipped with a filtered CuK radiation source and a luminous detector (step of 0.02° and counting time of 1 s/step).

On DSC Q200 equipment (TA Instruments, New Castle, DE, USA), differential scanning calorimetry (DSC) was performed in the temperature range of 0 to 380 °C with a heating rate of 10 °C/min under nitrogen flow.

The Easy Drop DSA20E Kruss GmbH equipment was used to measure the fibrous mats’ static water contact angle (Hamburg, Germany). After depositing a sessile drop of deionized water (10 μL in volume) onto the surface of the fibrous samples (2 cm × 7 cm, cut in the collector rotation direction), the average value of the contact angle was calculated using computer analysis. A total of 20 measurements per sample were made.

In order to determine the amount of quercetin and rutin in the fibrous materials, samples (1 cm^2^) were dissolved in 10 ml of an acetone/water solution. Then, using a DU 800 spectrophotometer, the absorbances of quercetin and rutin were detected at 371 and 257 nm, respectively (Beckman Coulter, CA, USA).

The release profile of quercetin and rutin was evaluated in vitro at 37 °C in an acetate buffer containing Tween 80 and having a constant ionic strength of 0.1 (CH_3_COONa/CH_3_COOH) at pH 5.5 (acetate buffer/Tween 80 = 99.2/0.8 *v*/*v*). The tested mats were soaked in a thermally controlled shaking water bath containing a 100 mL buffer solution that was stirred at 150 rpm (JULABO SW23, Allentown, PA, USA). At specified time points, aliquots of the test solution were taken out, and their absorbance was measured at 257 and 371 nm wavelengths. Using a calibration curve (correlation coefficient R = 0.999) for the mats in acetate buffer/Tween 80 (99.2/0.8 *v*/*v*), at pH = 5.5 and a constant ionic strength of 0.1, the quantity of released quercetin and rutin was determined.

Using a radical scavenging test with 2,2-diphenyl-1-picrylhydrazyl (DPPH), the antioxidant activity of the materials was determined. Ethanol solutions of QUE or RUT (5 × 10^−3^ M) or CA/PEG, CA/PEG/QUE, CA/PEG/RUT, CA/PEG/QUE/RUT, and CA/PEG/QUE+CA/PEG/RUT fibrous samples (0.5 mg) were immersed in 3 mL of DPPH solution in ethanol (1 × 10^−4^ M). All solutions were stored at 20 °C in the dark for 30 min. A DU 800 UV–VIS spectrophotometer (Beckman Coulter, CA, USA) was used to characterize the solutions’ absorbance at 517 nm in order to identify how many DPPH radicals were still present in the solution. The following equation was used to evaluate the antioxidant activity (AA%):

$$\mathrm{Inhibition},\;\mathrm{AA},\%=\left[\frac{(A_{\mathrm{DPPH}}-A_{\mathrm{sample}})}{A_{\mathrm{DPPH}}}\right]\times \;100\;$$

In the equation the A_sample_—DPPH• is the solution absorption at 517 nm after the addition of the flavonoid solution or fibrous materials and A_DPPH•_ is the absorption for DPPH• solution at 517 nm. Three duplicates of each experiment were carried out.

### 2.4. Cytotoxicity Assessment by MTT Cell Viability Assay

By using the MTT test, the impact of various fibrous materials on the viability of HeLa cells and mouse BALB/c 3T3 fibroblasts was evaluated [36]. In brief, the cultures were incubated in DMEM with 10% FBS, 100 U/mL penicillin, and 0.1 mg/mL streptomycin at 37 °C and 5% CO_2_ in a CO_2_ incubator. The cells were trypsinized with 0.25% Trypsin-EDTA once they had reached 80–90% confluence, and then they were counted using a hemocytometer. A 96-well microtiter plate was then used to retain the cells at a concentration of 1 × 10^5^ cells/well. HeLa and mouse fibroblasts were exposed to UV-sterilized fibrous mats (for 30 min) and cultured for 24 and 48 h after overnight incubation at 37 °C in a humidified environment containing 5% CO_2_.

As controls, the cell lines were cultured both alone and with QUE and RUT added. Five measurements were used to evaluate each variant. The HeLa and mouse fibroblasts were cultured with fibrous mats first, and then were twice washed with PBS (pH 7.4), followed by incubation at 37 °C for 3 h with 100 L of MTT working solution (Sigma Chemical). The supernatants were then removed, and 100 L of lysing solution (DMSO/ethanol 1:1) was added to each well to dissolve the resulting formazan. Using an ELISA plate reader (TECAN, Sunrise^TM^, Grodig/Salzburg, Austria), the MTT assay was detected.

Cell viability was defined as:


cell viability (%) = OD_570_ (experimental)/OD_570_ (control) × 100

### 2.5. Studying Apoptotic Induction Using AO and EtBr Dual Staining

Acridine orange (AO) and ethidium bromide (EtBr) were used to double-stain HeLa and mouse BALB/c 3T3 fibroblast cells in order to assess the cell death. The cells were plated on glass lamellas, placed at a concentration of 2 × 10^5^ cells × mL^−1^ at the bottom of 24-well plates, and incubated at 37 °C for 24 h in a CO_2_ incubator to form a monolayer. The fibrous samples were then sterilized using UV light and placed in 24-well plates for an additional 24 h of incubation. The fibrous materials were then removed, and glass lamellas were washed twice with phosphate-buffered saline (PBS, pH 7.4) to remove unattached cells. After that, the lamellas were stained with AO and EtBr at a ratio of 1:1 (10 μg/mL), and a fluorescence microscope (Leica DM 5000B, Wetzlar, Germany) was used to observe them.

### 2.6. DAPI Staining Assay

To examine the morphology of the cell nucleus, 4′,6-diamidino-2-phenylindole (DAPI) staining was employed as reported in [37]. This was accomplished by growing HeLa and mouse fibroblast cells (1 × 10^5^ cells/well) on glass cover slips in 24-well plates for 24 h in a CO_2_ incubator, followed by exposure to the fibrous materials. The cells were incubated, and then fixed with 3% paraformaldehyde at room temperature, followed by DAPI staining. The stained cells were then covered with 90% glycerol and observed under a fluorescence microscope (Leica DM 5000B, Wetzlar, Germany) to determine the nuclear morphology.

### 2.7. Statistical Analysis

The results’ data were displayed as means ± standard deviation (SD). One-way analysis of variance (ANOVA) and the post hoc comparison test (Bonferroni) were used with the GraphPad Prism program, version 5, to evaluate the statistical significance of the data (GraphPad Software Inc., San Diego, CA, USA). Statistics were considered to be significant for values of * *p* < 0.05, ** *p* < 0.01, and *** *p* < 0.001.

## 3. Results and Discussion

The fabrication of cellulose acetate defect-free fibers with a mean fiber diameter of 780 nm was achieved using electrospinning of solution with a concentration of 10 wt%. The release of relatively poorly water-soluble compounds such as curcumin [25] and quercetin [33] was facilitated by combining the CA polymer with the water-soluble polymer PEG. Therefore, fibrous CA/PEG polymer carriers are suitable for the incorporation of both hydrophilic and hydrophobic molecules of diverse origin, allowing effective drug protection and controlled drug release.

We hypothesize that the successful incorporation of QUE and RUT in fibrous polymer carriers will improve their bioavailability and cellular uptake in order to increase their therapeutic effectiveness.

### 3.1. Morphology of the Obtained Electrospun Mats

**Figure 1:**
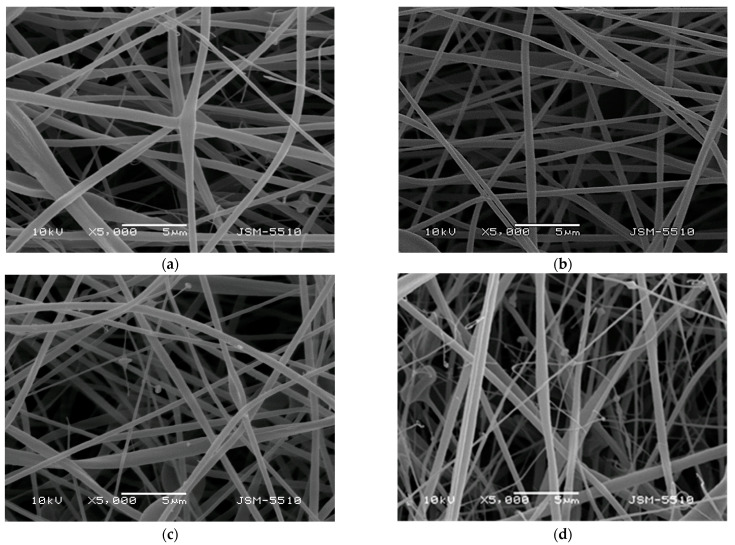

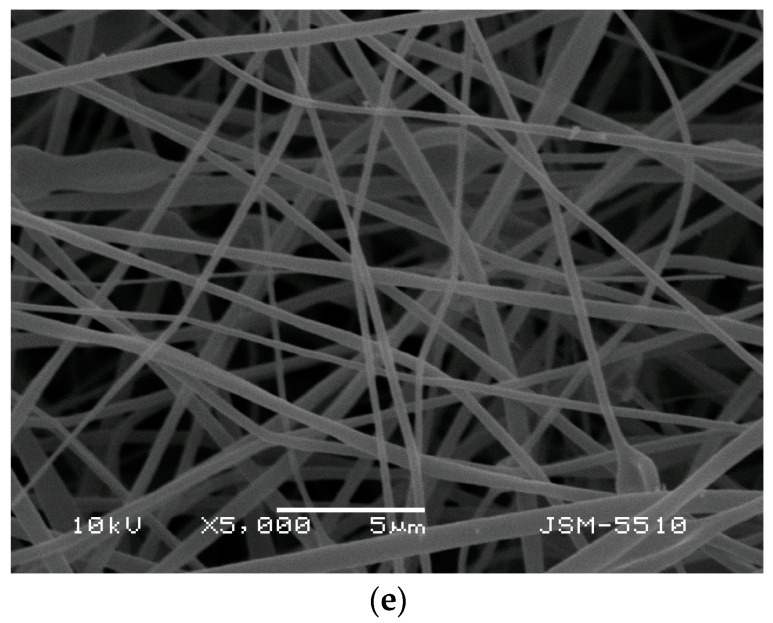
SEM images of the fibers based on: (**a**) CA/PEG, (**b**) CA/PEG/QUE, (**c**) CA/PEG/RUT, (**d**) CA/PEG/QUE/RUT, and (**e**) CA/PEG/QUE+CA/PEG/RUT.

Scanning electron microscopy (SEM) was used to analyze the morphology of all the obtained fibrous mats prepared by electrospinning. Figure 1 presents the fibrous morphology of the prepared materials from: (1) CA/PEG blend solution, (2) CA/PEG/QUE blend solution, (3) CA/PEG/RUT blend solution, (4) CA/PEG/QUE/RUT blend solution, and (5) CA/PEG/QUE solution+CA/PEG/RUT solution. The last fibrous material was obtained by simultaneous dual electrospinning from separate solutions of CA/PEG/QUE and CA/PEG/RUT in order to study the influence of different architecture. Figure 1a presents the SEM micrograph of the CA/PEG fibrous samples. As seen, CA/PEG fibers were with a cylindrical shape; however, some defects were detected. Defects were presented as a result of the addition of the low-molecular-weight, water-soluble polymer PEG, which reduced the viscosity of the solution. The measured mean fiber diameter of this type of fibers was 530 ± 150 nm. The addition of QUE or RUT to the spinning solution for CA/PEG led to the fabrication of the fibers with a mean diameter of 390 ± 100 and 375 ± 135 nm, respectively (Figure 1b,c). The electrospinning of a blend solution of CA/PEG/QUE/RUT resulted in the fabrication of fibers with a mean diameter of 366 ± 230 nm (Figure 1d). Dual electrospinning of solutions of CA/PEG/QUE and CA/PEG/RUT led to the obtaining of fibers with mean diameters of 385 ± 180 nm (Figure 1e). The detected reduction in fiber diameters is due to the slight decrease in the dynamic viscosity values of the flavonoid-containing solutions as compared with the CA/PEG solution.

### 3.2. ATR-FTIR Analysis

**Figure 2:**
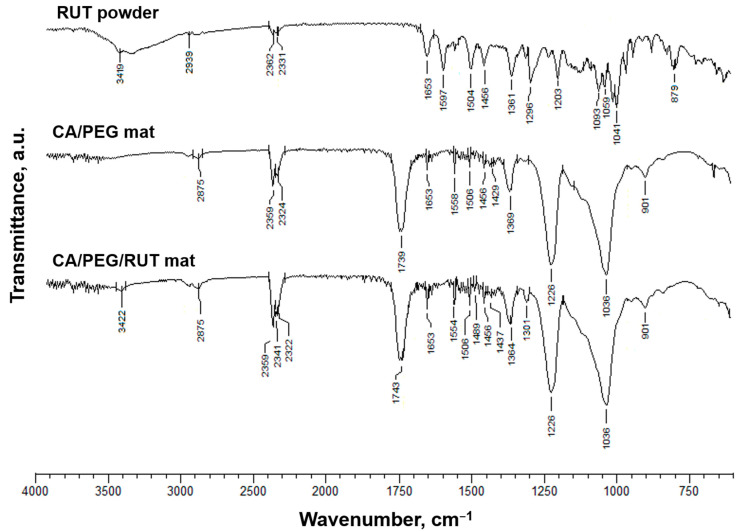
ATR-FTIR spectrum of rutin (powder), CA/ PEG fibers, and CA/ PEG/RUT fibers.

Using ATR-FTIR spectroscopy, CA/PEG, CA/PEG/QUE, CA/PEG/RUT, CA/PEG/QUE/RUT, and CA/PEG/QUE+CA/PEG/RUT fibrous samples were characterized. As shown in Figure 2, typical bands for the C=O functional groups at 1739 cm^−1^, for the CH_3_ groups at 1369 and 1226 cm^−1^, and for the ether C-O-C functional groups at 1037 cm^−1^ characteristic for the CA were found in the ATR-FTIR spectra of the CA/PEG mats [38]. Additionally, bands at 2875 cm^−1^ due to vC–H and at 1100 cm^−1^ characteristic of PEG ether groups were found. The main distinguishing bands for rutin were found to be hydroxyl (O-H) stretching at 3419 cm^−1^, carbonyl (C=O) stretching at 1653 cm^−1^, and benzene ring vibrations in the range of 1250–1600 cm^−1^ [39]. Rutin shows a characteristic carbonyl absorption band at 1653 cm^−1^, which is attributed to aromatic ketonic carbonyl stretching. At 1313 cm^−1^, a peak for the O-H functional group was also detected. Other distinguishing peaks were seen at 1059 cm^−1^, including C-O-C asymmetric stretching for ether and at 2939 cm^−1^ for alkane (CH_3_ asymmetric stretching) [40]. As shown in Figure 2, a shift in the characteristic band for C=O stretching vibrations up to 1743 cm^−1^ in comparison with the spectra of the native CA/PEG fibers (1739 cm^−1^) was seen in the ATR-FTIR spectrum of CA/PEG/RUT fibers. A shift at 1301 and 3422 cm^−1^ (for hydroxyl stretching) was detected. The hydrogen bonds between the CA or PEG molecules and the RUT molecules might be responsible for these shifts.

A shift in the characteristic band for C=O stretching vibrations to 1748 cm^−1^ was also observed in the CA/PEG/QUE fibers’ spectrum compared with the ATR-FTIR spectra of the CA/PEG fibers (1739 cm^−1^) (Figure S1a). Additionally, in the spectra of the CA/PEG/QUE mat, there is a distinct shift in the characteristic bands for C=C to 1600 and 1506 cm^−1^ compared with the spectrum of the quercetin powder (1606 and 1510 cm^−1^, respectively) [33]. Another shift for the band characteristic of the C=O of the aryl ketone groups of QUE from 1659 to 1653 cm^−1^ for the CA/PEG/QUE fibers was detected as well.

In the CA/PEG/QUE/RUT and CA/PEG/QUE+CA/PEG/RUT mats (Figure S1b), all these shifts are present as well, suggesting hydrogen bonding between CA, PEG, QUE, and RUT molecules.

### 3.3. Water Contact Angle

**Figure 3:**
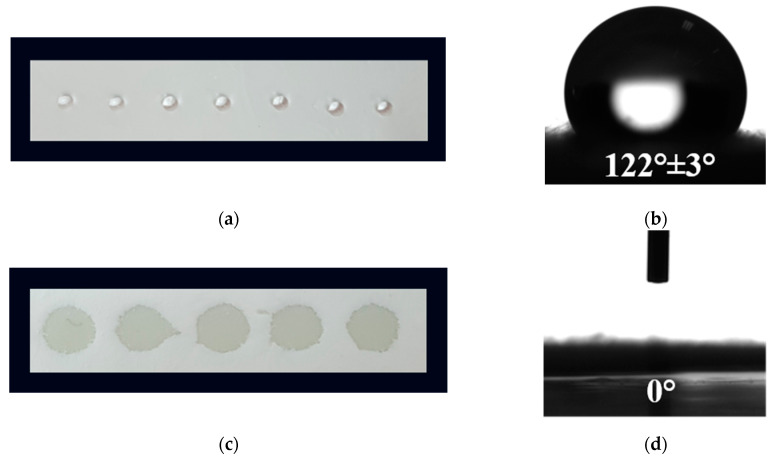
Digital photographs of distilled water drops (10 μL) placed on surfaces of: (**a**,**b**) CA mats and (**c**,**d**) CA/PEG/QUE/RUT mats.

The initial adherence of cells and their growth can be significantly altered by the hydrophilic/hydrophobic characteristics of the fibrous materials [41]. As a result, the water contact angles of the prepared fibrous mats were evaluated. Figure 3 displays digital images of the water droplets that were deposited on the fiber mats’ surfaces. In our previous study, we ascertained that the value of the water contact angle of the CA fibers is ~120° (Figure 3a,b) and that of the CA/PEG fibers is 0° [33]. In the present study, the measured value of the water contact angle of CA/PEG, CA/PEG/QUE, CA/PEG/RUT, CA/PEG/QUE/RUT, and CA/PEG/QUE+CA/PEG/RUT (Figure 3c,d) mats was 0°, and the water drop was immediately absorbed by the fibrous material. Due to the inclusion of the water-soluble polymer, PEG, which improves the materials’ wettability, the resulting electrospun materials were hydrophilic.

In particular, when considering the potential biological application of these fibrous materials in the local treatment of cervical cancers, their imparted hydrophilicity is a crucial characteristic for attaining a rapid therapeutic action of the biologically active substances incorporated in the material.

### 3.4. X-ray Diffraction Studies

**Figure 4:**
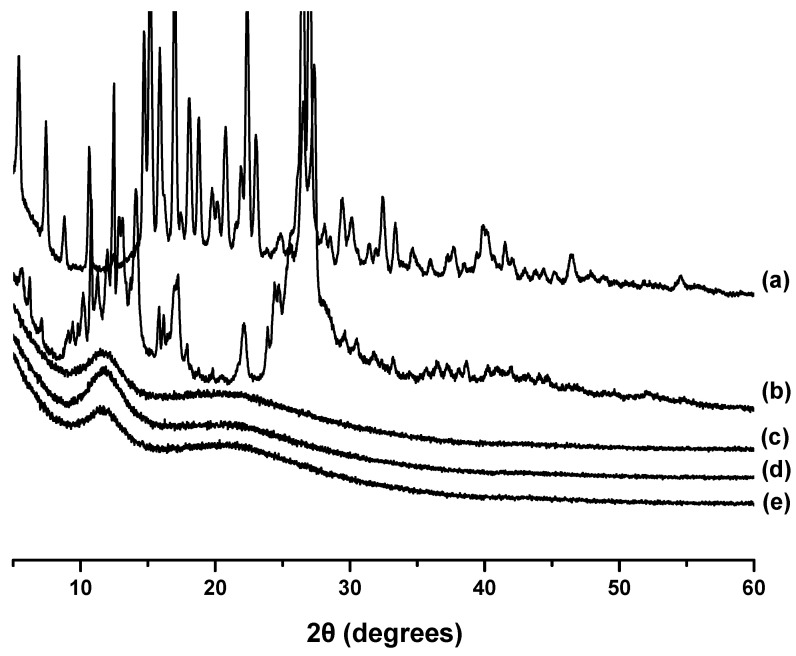
XRD patterns of: (**a**) rutin (powder), (**b**) quercetin (powder), (**c**) CA/PEG/RUT mat, (**d**) CA/PEG/QUE/RUT mat, and (**e**) CA/PEG/QUE+CA/PEG/RUT mat.

In our previous study, we proved that the electrospun CA/PEG and CA/PEG/QUE mat were in amorphous state [33]. Figure 4 presents the XRD patterns of RUT (powder), QUE (powder), CA/PEG/RUT mat, CA/PEG/QUE/RUT mat, and CA/PEG/QUE+CA/PEG/RUT mat recorded in the range of 2θ 10–60°. In the X-ray diffractogram of rutin powder (Figure 4a), significant and distinct crystallinity peaks at 2θ = 11.2°, 15.5°, 16.5°, and 23 ° are detected, suggesting its crystalline nature. This finding was consistent with that of Ravi et al. [42]. The XRD pattern of quercetin powder showed a highly crystalline nature as evident from the sharp peaks observed at 12.5°, 15.7°, 17.3°, and 27.3°. These sharp diffraction peaks showed that QUE (powder) is highly crystalline as well.

Furthermore, X-ray diffraction studies revealed that the CA/PEG mats containing QUE and/or RUT are amorphous. The XRD patterns of all electrospun fibrous materials present the existence of an amorphous halo, as shown in Figure 4c–e. The absence of diffraction peaks for QUE and RUT’s crystalline phases indicates that the biologically active substances incorporated into the fibers were in an amorphous form.

### 3.5. Thermal Analysis

**Figure 5:**
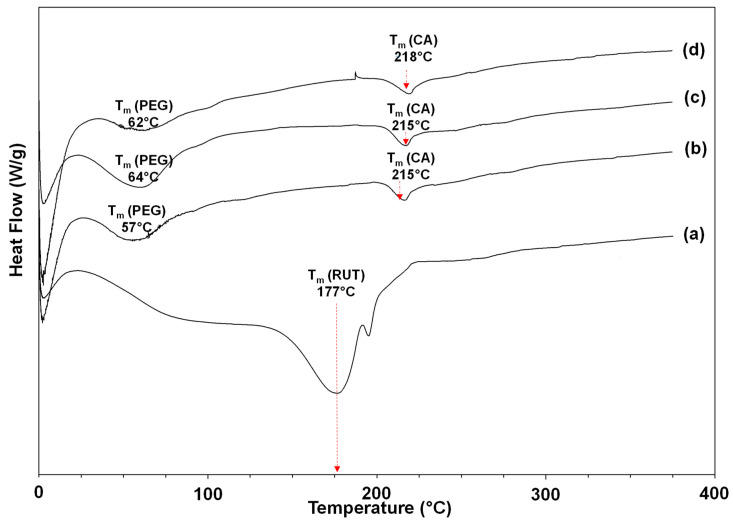
DSC thermograms of: (**a**) rutin (powder), (**b**) CA/PEG/RUT mat, (**c**) CA/PEG/QUE/RUT mat, and (**d**) CA/PEG/QUE+CA/PEG/RUT mat.

The thermal behavior of the used flavonoids (powder) and of the obtained novel fibrous materials was determined by DSC. The applied temperature range was from 0 to 380 °C. The thermograms of RUT-containing mats and RUT (powder) are shown in Figure 5, while the thermograms of QUE-containing mats and QUE (powder) are presented in Supplementary Material, Figure S2. In the thermogram of RUT (powder), a melting peak at 177 °C was detected (Figure 5a). The observed peak is in good accordance with data from the literature that describe a DSC curve of unprocessed rutin at 174 °C that corresponds to flavonoid’s phase transition [43]. A melting peak for QUE (powder) appeared at 323 °C during the first heating cycle (Supplementary Material, Figure S2), along with a wide endothermic peak (116 °C) attributed to the loss of water molecules. The CA/PEG fibrous mat exhibits a peak for the PEG melting point at 57 °C and an endothermic peak for the Tm of cellulose acetate at 209 °C (Supplementary Material, Figure S2). No peaks matching the melting points of QUE and RUT were observed in the DSC thermogram of the CA/PEG/QUE, CA/PEG/RUT, CA/PEG/QUE/RUT, and CA/PEG/QUE+CA/PEG/RUT mats. According to this result, the QUE and RUT that were loaded into the fibers were amorphous. Additionally, this result is consistent with the XRD analysis results.

### 3.6. In Vitro Release Testing Methods

Generally, flavonoids’ solubility is very low in water [44]. The aqueous solubility of rutin is 0.125 g/L, and that of quercetin is 0.00215 g/L [45]. Therefore, it is necessary to develop formulations capable of increasing the water solubility of these compounds, improving their bioavailability and, consequently, their biological activity. The composition of the polymer matrix in which the biologically active compounds were incorporated will have a significant role in the release profile. In the current research for improving the water wettability and, thus, the release of QUE and RUT, the polymer matrix purposefully contains a water-soluble polymer with low molecular weight.

Another major factor affecting the release rate from polymer matrixes is the crystallinity of the used polymer and active substances. Amorphous compounds are generally more soluble and faster dissolving than their stable crystalline counterparts due to higher free energy and greater molecular mobility.

**Figure 6:**
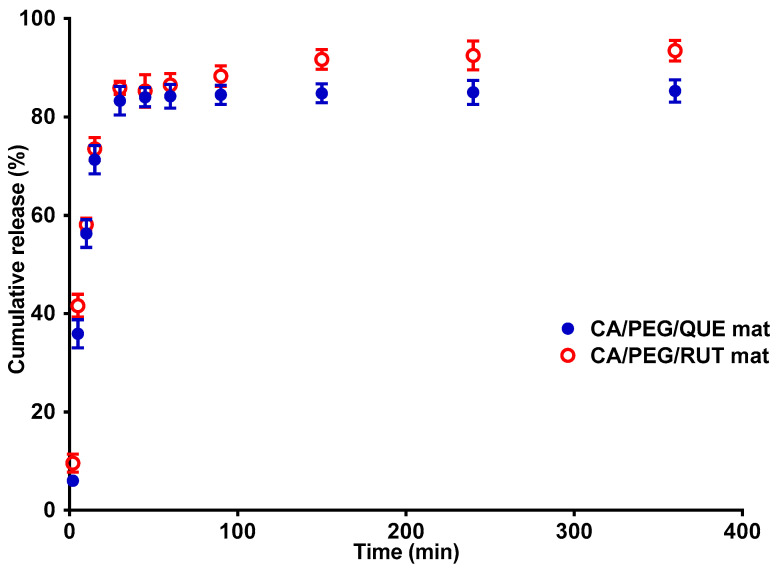
Release profiles of QUE and RUT from CA/PEG/QUE and CA/PEG/RUT fibrous mats. The data are displayed as average values from three different measurements along with their standard deviations, volume ratio of acetate buffer to Tween 80 (99.2:0.8 *v*/*v*), ionic strength of 0.1, pH 5.5, 37 °C.

The in vitro release profile of the plant-derived compounds: the model flavonoid RUT and QUE, from cellulose acetate and PEG polymer fibrous matrix was followed. In order to evaluate the impact of matrix composition on the release profile of both flavonoids, the release was spectrophotometrically monitored in acetate buffer (pH 5.5, ionic strength of 0.1) in the presence of Tween 80 at a ratio of the volume of acetate buffer to Tween 80: 99.2:0.8 (at 37 °C). Tween 80 is a nonionic surfactant often used with poorly soluble compounds to improve their solubility and increase their absorption. The results from the QUE and RUT release from the CA/PEG/QUE and CA/PEG/RUT electrospun material are presented in Figure 6. RUT was found to be rapidly released from the fibrous mats compared with the release of QUE for the same time period. Approximately 93.5% of RUT was released from the CA/PEG/RUT fibrous materials for 360 min, compared with 85.3% of the QUE amount.

The flavonoid release from the complex fibrous materials is presented in Supplementary Material, Figure S3. The released QUE and RUT amounts from the CA/PEG/QUE/RUT mat and CA/PEG/QUE+CA/PEG/RUT mat are comparable to the release of the individual flavonoids from the CA/PEG materials. The detected released amount of QUE and RUT was insignificantly less from the mat based on CA/PEG with both QUE and RUT loaded. This is most probably due to the more complex architecture of the electrospun material.

The CA/PEG/RUT, CA/PEG/QUE, CA/PEG/QUE/RUT, and CA/PEG/QUE+CA/PEG/RUT mats are hydrophilic with contact angle values of 0°, which assists the medium molecules to penetrate into the fibrous materials. Generally, this is a prerequisite for the formation of a water-soluble complex based on hydrogen bonds between the used flavonoids and PEG, which facilitates the QUE and RUT release.

### 3.7. Determination of Antioxidant Properties

**Figure 7:**
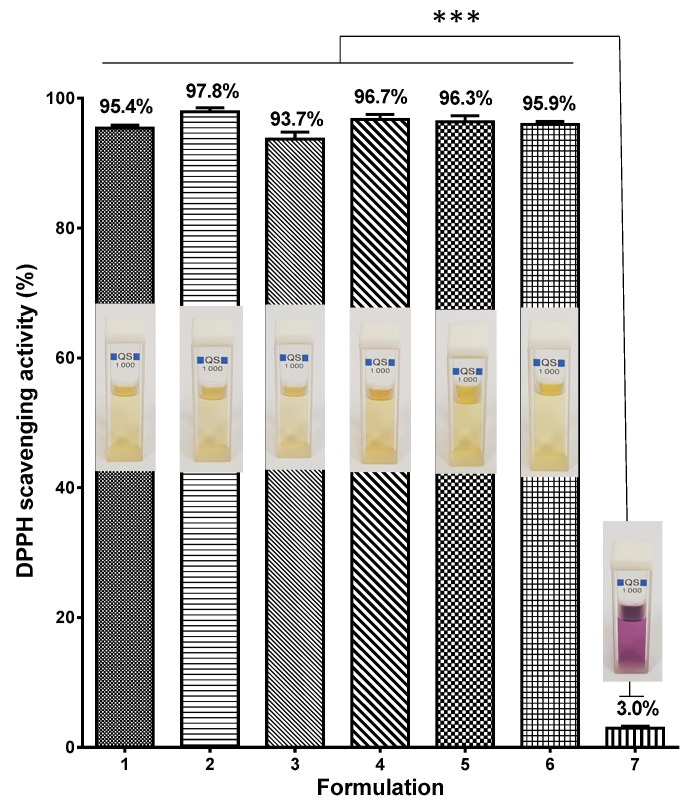
Antioxidant activity of: (1) ethanol solution of QUE, (2) ethanol solution of RUT, (3) CA/PEG/QUE fibers, (4) CA/PEG/RUT fibers, (5) CA/PEG/QUE/RUT fibers, (6) CA/PEG/QUE+CA/PEG/RUT fibers, (7) CA/PEG fibers. *** *p* < 0.001. Photos of the corresponding solutions are presented as digital images.

It has been demonstrated that quercetin and rutin reveal antioxidant properties [11,46]. The DPPH radical scavenging activity of CA/PEG mats containing QUE and/or RUT was detected. The absorbance of a DPPH radical dot at 517 nm 30 min after contact with fibrous mats was measured spectrophotometrically. The DPPH scavenging capacity of CA/PEG fiber mats was also determined for comparison. According to Figure 7, the CA/PEG fibers displayed a negligible impact on the DPPH solutions (the absorbance of the DPPH radical dot dropped by about 3.0%, as shown in Figure 7(7)). Additionally, there was no significant change in the color of the DPPH solution in contact with the CA/PEG fibers. In contrast, mats loaded with QUE and/or RUT revealed strong antioxidant activity after 30 min of exposure to the DPPH solution, as can be seen in Figure 7(1–6). Upon contact with CA/PEG mats loaded with QUE and/or RUT, the DPPH solution’s color changed to pale yellow. Furthermore, the change in DPPH solution absorption after contact with an ethanol solution of flavonoids is comparable to the result of the contact with the fibrous mat incorporated with QUE and/or RUT at the same concentration of biologically active molecules. It is observable that the fibrous materials containing RUT or QUE/RUT exhibited slightly higher antioxidant activity than the mats containing QUE. All these findings reveal that both QUE and RUT retained their high antioxidant capacity upon loading in CA/PEG fibrous materials.

### 3.8. Cytotoxicity Studies against Human HeLa Tumor Cells and BALB/c 3T3 Fibroblasts

**Figure 8:**
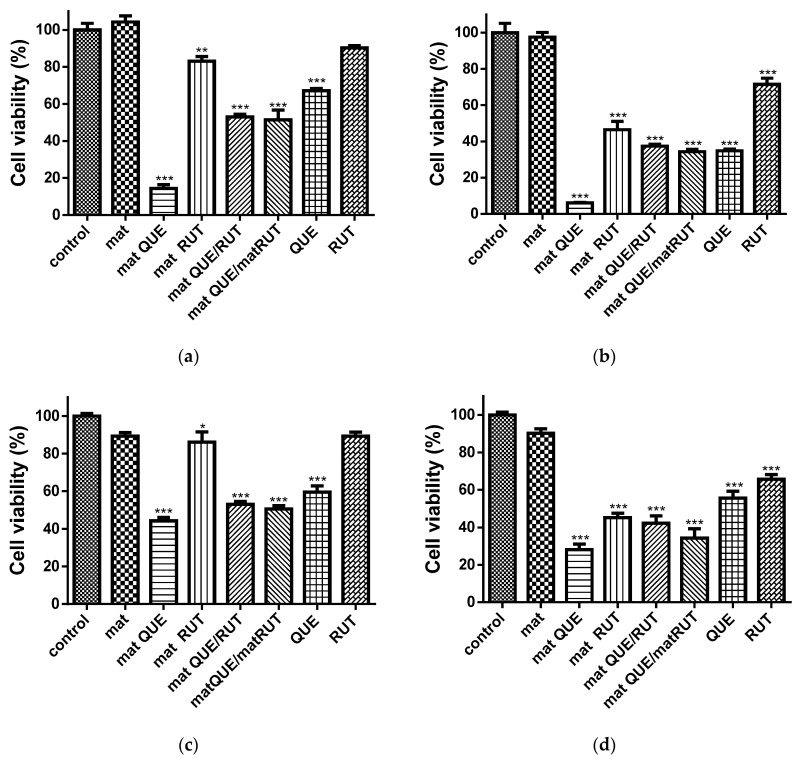
Effect of mats on HeLa tumor cells (**a**,**b**) and mouse BALB/c 3T3 fibroblast cells (**c**,**d**) after 24 h (**a**,**c**) and 48 h (**b**,**d**) of incubation with different formulations: control—untreated HeLa cells or mouse BALB/c 3T3 fibroblast cells, CA/PEG mat, CA/PEG/QUE mat, CA/PEG/RUT mat, CA/PEG/QUE/RUT mat, CA/PEG/QUE+CA/PEG/RUT mat, QUE (50 µM/L), RUT (50 µM/L), * *p* < 0.05, ** *p* < 0.01. and *** *p* < 0.001.

Recently, there has been great global interest and a rising demand for effective, low-toxicity, new anticancer agents. Rutin and quercetin have been found to be cytotoxic to a variety of cancer cells [18,47]. In the current study, the cytotoxic effects of the obtained fibrous mats were assessed using the MTT test on human cervical HeLa cells and BALB/c 3T3 fibroblasts for 24 and 48 h. The results are presented in Figure 8. The results show a statistically significant inhibition of the proliferation of the two types of investigated cells in the presence of the fibrous mats containing QUE and/or RUT at both time intervals, with the reported values at 48 h being lower than those at 24 h. In both cell types, the control mat of CA/PEG was nontoxic, and cell viability was close to that of the control cells. The lowest viability was observed when cells were cultured with the CA/PEG/QUE mat. The values for BALB/c 3T3 fibroblasts were 44.2% ± 3.4% at 24 h and 28.1% ± 6.5% at 48 h. In HeLa tumor cells, the determined values at the same time intervals were 3 to 4.5 times lower—14.3% ± 4.6% and 6.0% ± 0.9%, respectively. The CA/PEG/RUT, CA/PEG/QUE/RUT, and CA/PEG/QUE+CA/PEG/RUT mats significantly reduced the viability of both cell lines, with a slightly stronger inhibition being detected in HeLa tumor cells. When 3T3 fibroblasts were cultured in the presence of CA/PEG/QUE/RUT or CA/PEG/QUE+CA/PEG/RUT fibrous samples, viability was reduced to 50% and 52% at 24 h, respectively, and to 42% and 34% at 48 h. Similar values were measured when cultivating cancer cells with the same fibrous mats—respectively, 52% and 51% at 24 h and 37% and 34% at 48 h. A higher viability was observed when both cell lines were cultured with CA/PEG/RUT fibrous material with values of 86.0% ± 9.0% and 45.1% ± 5.1% at 24 and 48 h for the fibroblast line and values of 83.0% ± 4.9% and 46.4 % ± 10.2% at 24 and 48 h for the cancer cells. The mats containing QUE displayed higher cytotoxicity compared with RUT-containing mats. Nevertheless, the QUE or QUE/RUT-containing mats displayed strong antiproliferative activity on cancer cells while exhibiting low toxicity against normal cells.

### 3.9. Analysis of Cell Death by Staining Methods

**Figure 9:**
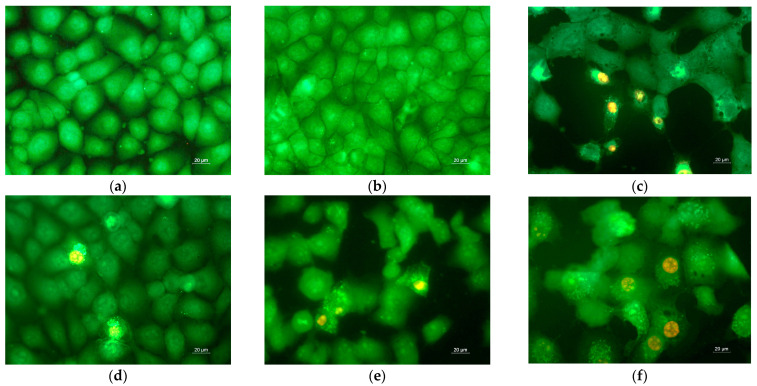
Fluorescence images of AO and EtBr double-stained HeLa cancer cells incubated for 24 h: (**a**) untreated cells and after incubation with (**b**) CA/PEG fibrous mat, (**c**) CA/PEG/QUE fibrous mat, (**d**) CA/PEG/RUT fibrous mat, (**e**) CA/PEG/QUE/RUT fibrous mat, and (**f**) CA/PEG/QUE+CA/PEG/RUT fibrous mat; bar = 20 μm.

Studies have been carried out to determine the extent to which the inhibition of HeLa cell proliferation is achieved via apoptosis. HeLa cells were grown for 24 h in the presence of the obtained fibrous mats before being stained with a combination of fluorescent dyes (AO and EtBr) (1:1 *w*/*w*). The cells were stained and examined under a fluorescence microscope (Figure 9). Morphological analysis of control HeLa tumor cells and HeLa cultured with a CA/PEG fibrous sample showed the presence of green-stained cells normal in shape and nuclear structure, as well as the presence of cells in the division phase (Figure 9a,b). The cells cultured with CA/PEG/RUT fibrous material were slightly rounded, and a thinning of the cell monolayer was observed (Figure 9d). Cancer cells cultured in the presence of the CA/PEG/QUE mat, CA/PEG/QUE/RUT mat, and CA/PEG/QUE+CA/PEG/RUT mat had changed shape, had impaired monolayer growth, and showed obvious alterations in the morphology of the cells and their nucleus. Additionally, cell number reduction was observed (Figure 9c,e,f). Severe alternations and morphological changes were observed in the tumor cells after their contact with CA/PEG/QUE (Figure 9c), the CA/PEG/QUE/RUT mat (Figure 9e), and the CA/PEG/QUE+CA/PEG/RUT mat (Figure 9f). Cells with late-apoptotic changes predominate—with blebbing of the cell membrane, condensation and margination of chromatin and fragmentation of the nucleus. The presence of apoptotic bodies was detected as well. According to the results, QUE-containing mats had a strong cytotoxic effect on HeLa cells; however, RUT-containing mats showed weaker cytotoxicity.

Furthermore, HeLa cell nuclei were examined for alterations using the DAPI staining method. The nuclei of the HeLa control exhibited intact nuclei, a quite oval shape, consistent size, smooth edges, and equally dispersed chromatin and were uniformly stained (Supplementary Material, Figure S4). Changes in the nuclei of HeLa tumor cells cultured in the presence of fibrous mats and stained with DAPI corresponded to the changes described after staining of the cells with AO and EtBr. The most severe damage was observed after culturing of cells with the CA/PEG/QUE mat, followed by the CA/PEG/QUE/RUT and CA/PEG/QUE+CA/PEG/RUT fibrous mats. Moderate alternations were observed after contact with the CA/PEG/QUE/RUT mat.

When normal mouse fibroblasts were cultured with mats, the observed morphological changes in cells were similar. Fibroblast control and fibroblasts cultured with the CA/PEG mat were green-stained, with a shape and morphology characteristic for this cell type (Supplementary Material, Figure S5a,b). Cells with early apoptotic changes and single cells with signs of late apoptosis predominated when fibroblasts were in contact with the QUE- and RUT-containing mats. The cells cultured with CA/PEG/QUE were the most affected. However, the monolayer growth was not impaired (Supplementary Material, Figure S5). The performed experiments with HeLa cancer cells and with normal fibroblast cells indicated that quercetin- and rutin-containing CA/PEG fibrous mats caused cell death by apoptosis. The combined strong antioxidant activity and in vitro anticancer characteristics render CA/PEG fibrous materials loaded with QUE and RUT suitable candidates for use as wound dressings and in the local treatment of cervical cancers.

## 4. Conclusions

Novel CA/PEG-based fibrous materials containing flavonoids—QUE and RUT—were successfully created by the electrospinning method. The developed electrospun mats were hydrophilic with a water contact angle of 0°. RUT released from the CA/PEG/RUT fibrous materials was around 93.5% for 360 min, whereas QUE released was 85.3% for the same time period. Fibrous materials containing RUT displayed strong antioxidant activity. Additionally, the flavonoid-containing mats exhibited good cytotoxicity against cancer cells due to the induction of apoptosis. The most prominent was the cytotoxicity of the CA/PEG/QUE mats. These characteristics show that the prepared novel fibrous materials have potential for use as local cervical tumor treatments and in wound dressing applications.

## Data Availability

Not applicable.

## Supplementary Materials

The following supporting information can be downloaded at: https://www.mdpi.com/article/10.3390/polym14245380/s1, Figure S1: ATR-FTIR spectra of (a) quercetin powder, CA/PEG, and CA/PEG/QUE fibers and (b) CA/PEG/QUE/RUT and CA/PEG/QUE+CA/PEG/RUT fibrous mat. Figure S2. DSC thermograms of: quercetin (powder), CA/PEG/QUE fibrous mat, and CA/PEG fibrous mat. Figure S3. QUE and RUT release profile from CA/PEG/QUE/RUT and CA/PEG/QUE+CA/PEG/RUT mats. The data are displayed as average values from three different measurements along with their standard deviations; volume ratio of acetate buffer to Tween 80 (99.2:0.8 *v*/*v*), ionic strength of 0.1, pH 5.5, 37 °C. Figure S4. Fluorescence micrographs of DAPI-stained HeLa cancer cells incubated for 24 h with fibrous mats. (a) Untreated cells and HeLa cells after incubation with: (b) CA/PEG mat, (c) CA/PEG/QUE mat, (d) CA/PEG/RUT mat, (e) CA/PEG/QUE/RUT mat, and (f) CA/PEG/QUE+CA/PEG/RUT mat. Bar = 20 µm. Figure S5. Fluorescence images of AO and EtBr double-stained BALB/c 3T3 fibroblast cells incubated for 24 h: (a) untreated fibroblasts and after incubation with: (b) CA/PEG mat, (c) CA/PEG/QUE mat, (d) CA/PEG/RUT mat, (e) CA/PEG/QUE/RUT mat, and (f) CA/PEG/QUE+CA/PEG/RUT mat; bar = 20 μm.
