# Supplementary material for: Quercetin- and Rutin-Containing Electrospun Cellulose Acetate and Polyethylene Glycol Fibers with Antioxidant and Anticancer Properties
Source: Polymers (Basel). 2022 Dec 8;14(24):5380. doi: 10.3390/polym14245380 (PMC9783884; doi:10.3390/polym14245380)
Supplement: Supplementary file 1 [file polymers-14-05380-s001.zip › polymers-2073129-supplementary.pdf]

Supplementary material

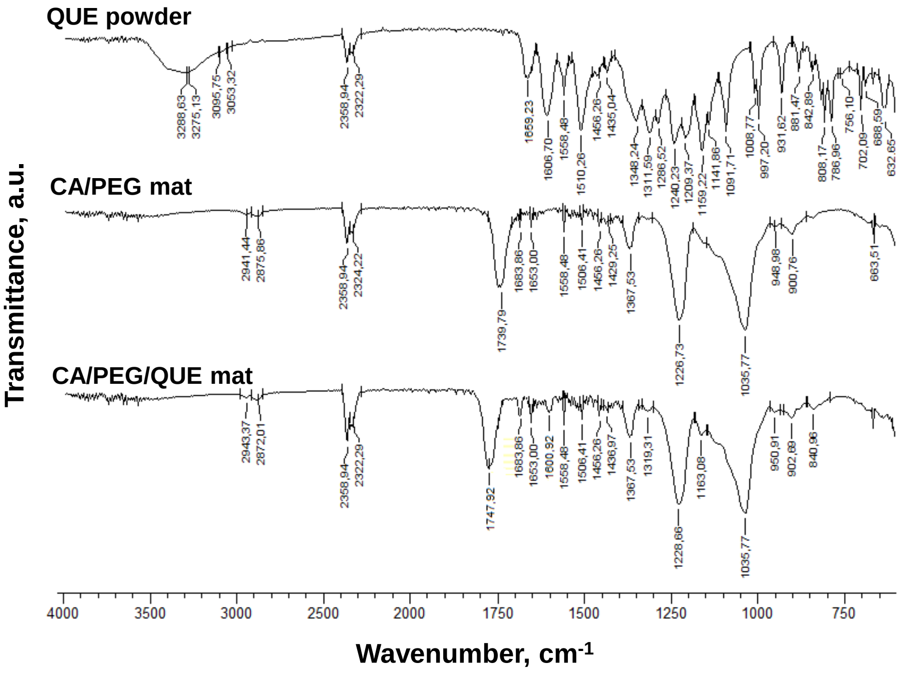

(**a**)

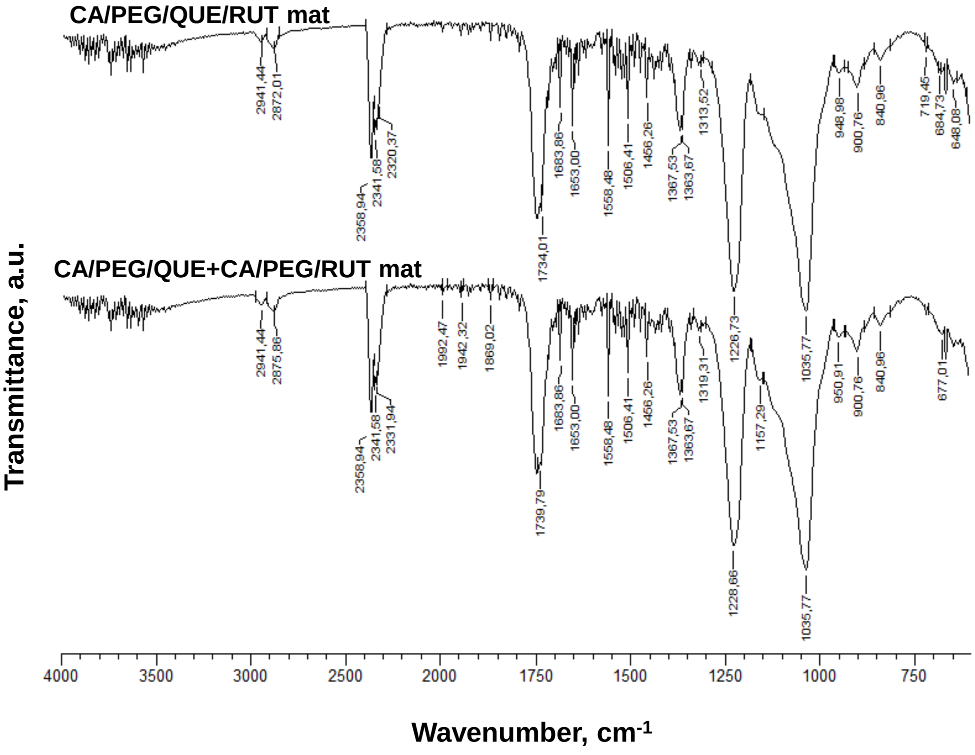

(**b**)

Figure S1: ATR-FTIR spectra of (a) Quercetin powder, CA/PEG and CA/PEG/QUE fibers and (b) CA/PEG/QUE/RUT and CA/PEG/QUE+CA/PEG/RUT fibrous mat.

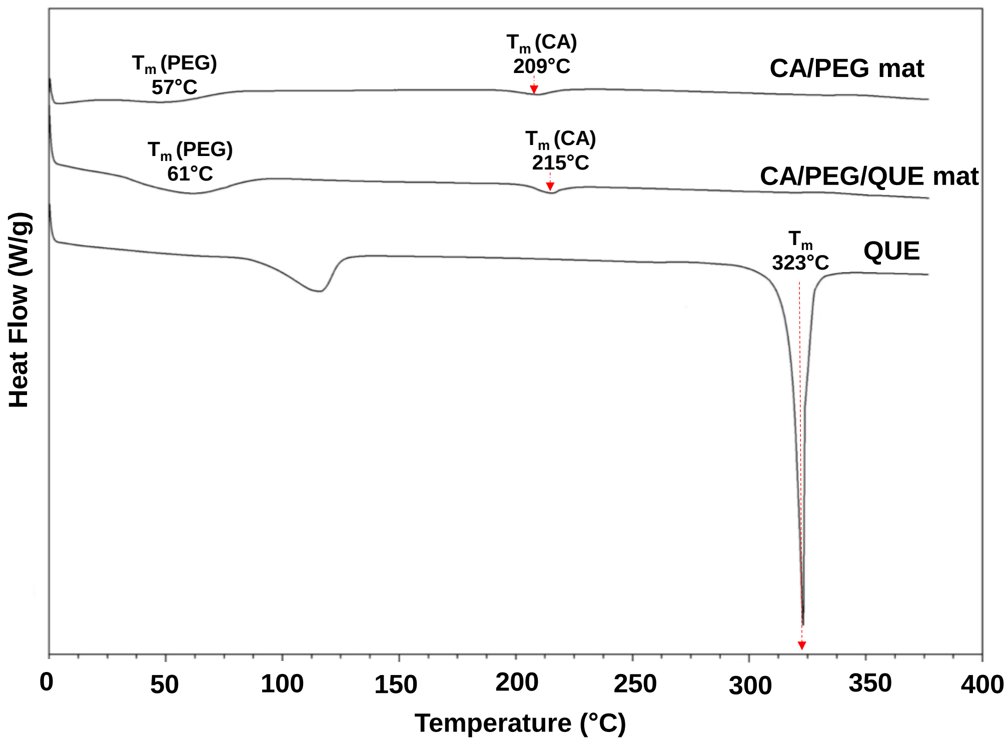

Figure S2. DSC thermograms of: quercetin (powder), CA/PEG/QUE fibrous mat, and CA/PEG fibrous mat.

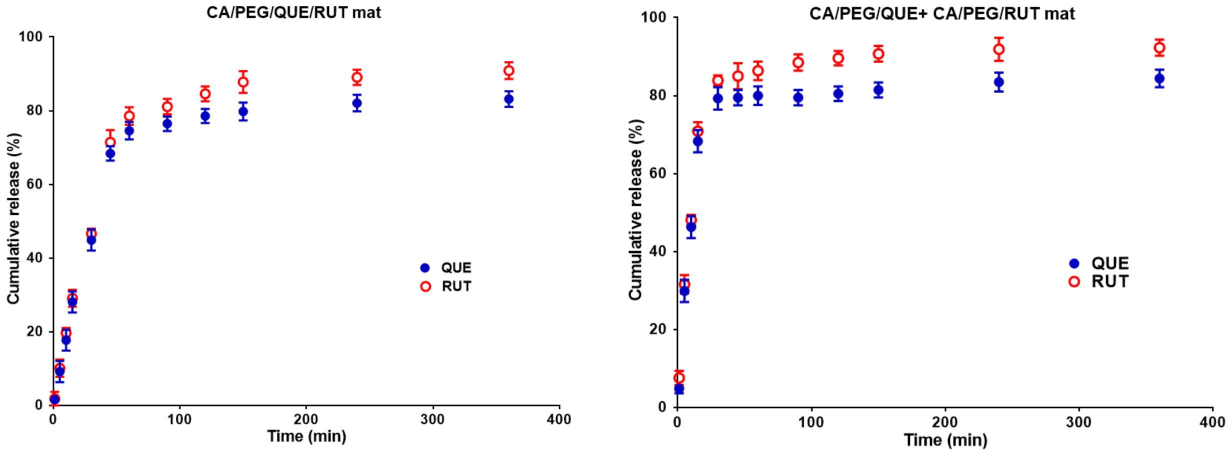

(**a**) (**b**)

Figure S3. QUE and RUT release profile from CA/PEG/QUE/RUT and CA/PEG/QUE+CA/PEG/RUT mats. The data are displayed as average values from three different measurements along with their standard deviations; volume ratio of acetate buffer to Tween 80 (99.2/0.8 v/v), ionic strength 0.1, pH 5.5, 37°C.

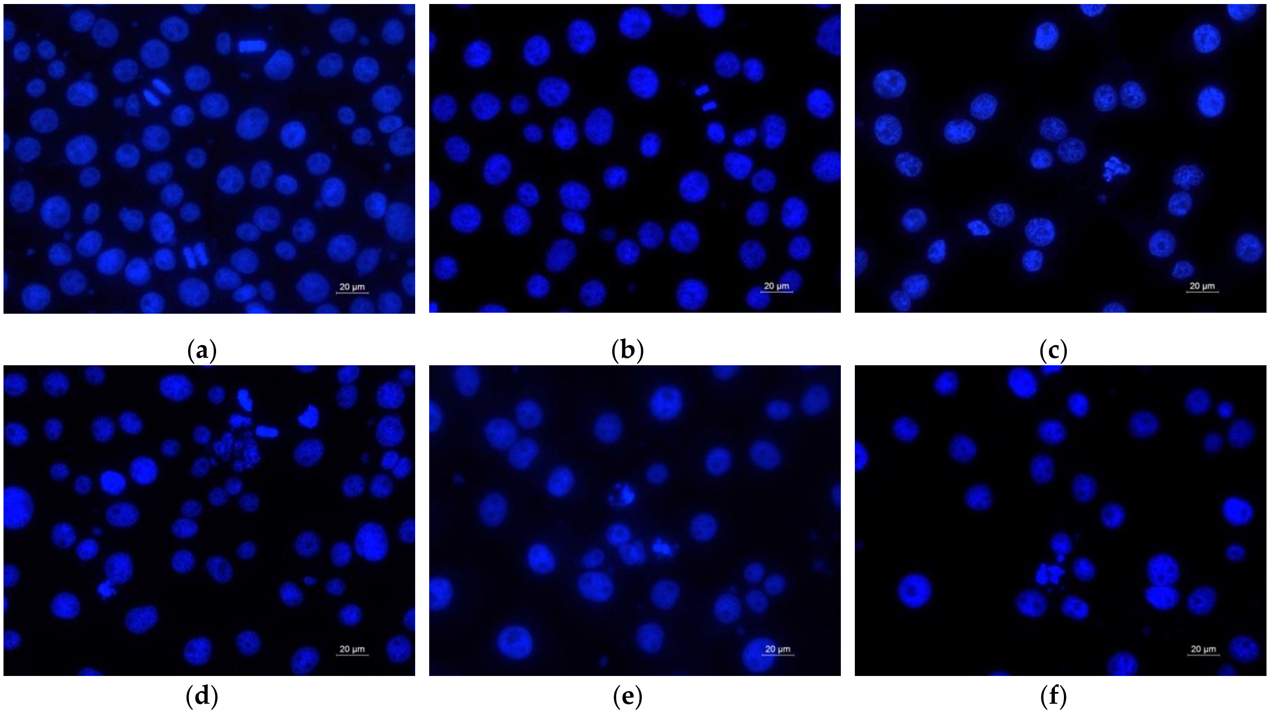

Figure S4. Fluorescence micrographs of DAPI-stained HeLa cancer cells incubated for 24 h with fibrous mats. (a) Untreated cells and HeLa cells after incubation with: (b) CA/PEG mat, (c) CA/PEG/QUE mat, (d) CA/PEG/RUT mat, (e) CA/PEG/QUE/RUT mat and (f) CA/PEG/QUE+CA/PEG/RUT mat. Bar =20μm.

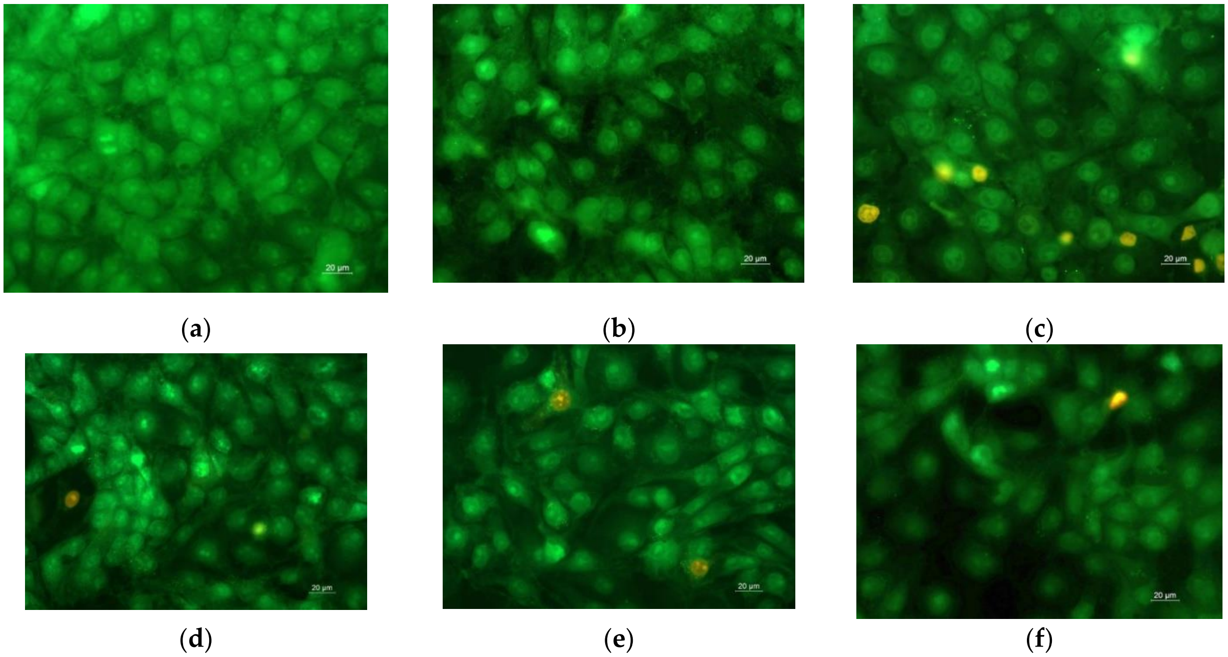

Figure S5. Fluorescence images of AO and EtBr double-stained Balb/c3T3 fibroblast cells incubated for 24 h (a) untreated fibroblasts and after incubation with: (b) CA/PEG mat, (c) CA/PEG/QUE mat, (d) CA/PEG/RUT mat, (e) CA/PEG/QUE/RUT mat and (f) CA/PEG/QUE+CA/PEG/RUT mat; bar = 20 μm.
